# Posterior Reversible Encephalopathy Syndrome (PRES) and Takotsubo: A Heart and Brain Affair!

**DOI:** 10.7759/cureus.13452

**Published:** 2021-02-20

**Authors:** Talha Perwez, Ahsan Wahab, Zunirah Ahmed, Aqsa Khan, Zahoor Ahmed

**Affiliations:** 1 Internal Medicine, University of Alabama at Birmingham (UAB), Montgomery, USA; 2 Internal Medicine Department, Baptist Medical Center South, Montgomery, USA; 3 Gastroenterology, Methodist Health System, Houston, USA; 4 Medicine, Fatima Jinnah Medical University, Lahore, PAK; 5 Internal Medicine, King Edward Medical University, Mayo Hospital, Lahore, PAK

**Keywords:** pres, takotsubo cardiomyopathy, stress, catecholamines

## Abstract

Takotsubo cardiomyopathy (TC) is characterized by reversible left ventricle systolic dysfunction usually associated with stressors (physiological, psychological) being triggering factors. The increase in sympathetic activity, along with a subsequent surge of catecholamines, has been hypothesized as a possible etiology of TC. Posterior reversible encephalopathy syndrome (PRES), a relatively rare and recently recognized reversible clinico-radiological syndrome, is thought to share the same pathophysiology as TC. We present a case of an 83-year-old female who presented with seizures and was found to have PRES. Within three days of hospitalization, she developed takotsubo. She endorsed being under significant emotional stress that was thought to be the common culprit for both of her syndromes, i.e., PRES and TC.

## Introduction

Takotsubo cardiomyopathy (TC), also known as stress cardiomyopathy or broken heart syndrome is specified by reversible left ventricle systolic dysfunction in the absence of influential coronary occlusions. TC mimics the acute coronary syndrome (accounts for 2% of all acute coronary syndromes), causes wall motion abnormalities, but lacks the obstructive coronary pathophysiology [1]. Many emotional or physical triggers have been proposed to induce sympathetic overactivity, causing a surge of catecholamines responsible for TC. Posterior reversible encephalopathy syndrome (PRES), a relatively rare and recently recognized reversible clinico-radiological syndrome, is characterized by acute neurologic changes, radiological evidence of focal vasogenic edema (predominantly parieto-occipital lobes), and the reversibility of clinical and/or imaging findings [2]. Many pathophysiological triggers of PRES such as uncontrolled hypertension, cytotoxic drugs, sepsis, and eclampsia are speculated to impair the blood-brain barrier, disrupt cerebral perfusion autoregulation, and lead to cerebral vascular leakage [2]. Both TC and PRES are suggested to share similar triggers or common pathophysiology [1-2]. Our case highlights the association between these two syndromes, suggesting a common etiological pathway.

## Case presentation

**Figure 1:**
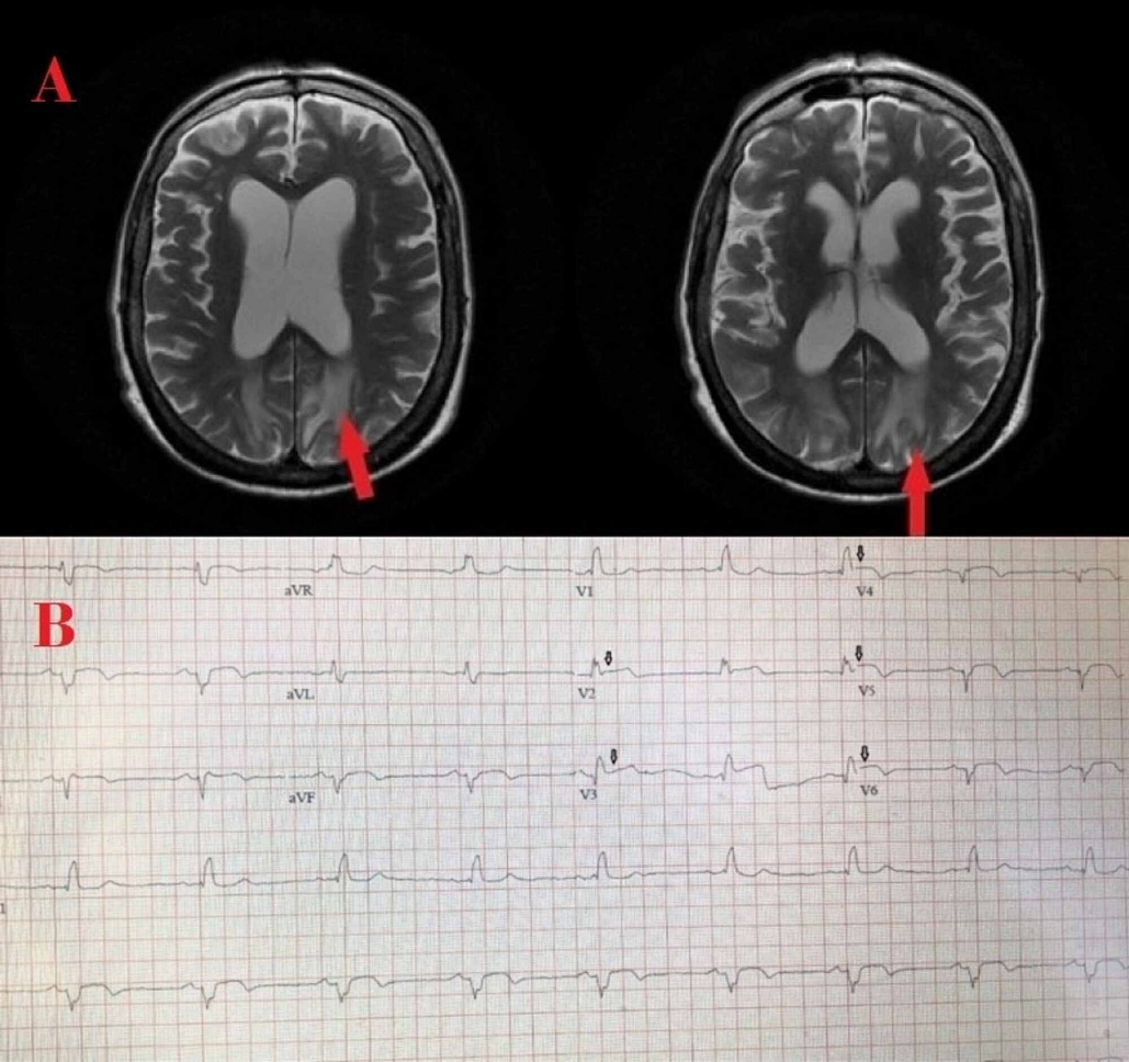
The magnetic resonance imaging (MRI) and electrocardiographic (EKG) findings of patients with posterior reversible encephalopathy syndrome (PRES) and takotsubo A) MRI of the brain showing hyperintense T2 signals (arrows) in the periventricular and subcortical white matter of both cerebral hemispheres. B) EKG showing ST-segment elevation in leads V2-V6 (arrows).

An 83-year-old Caucasian female with a history of diabetes mellitus, hypertension, and cerebrovascular accident presented to the emergency department with new-onset generalized tonic-clonic seizures, witnessed by her daughter. Upon arrival, she had another similar seizure, after which she got obtunded and had post-ictal confusion. Her vitals were temperature: 99.2 ºF, pulse: 102 beats/minute, blood pressure: 198/100 mm/Hg, respirations: 16 breaths/minute, and oxygen saturation (SpO2): 98% on 2 liters of oxygen via nasal cannula. At the time of our evaluation (which was approximately three hours after presentation), the physical exam was normal without focal neurological deficits. Computed tomography (CT) of the brain was not significant for acute changes. Magnetic resonance imaging (MRI) (Figure 1, panel A) revealed diffuse hyperintense T2-signals in the periventricular and subcortical white matter along with two small foci of restricted diffusion consistent with punctate infarcts involving the high parietal lobe and middle left occipital lobe. The diagnosis of PRES with seizures was made. She was started on intravenous levetiracetam therapy and blood pressure was optimized. No further seizures were recorded. The patient was reported to be extremely stressed out lately due to the death of her only brother, which was thought to be contributing to elevated blood pressure and PRES.

On the third day of admission, she reported persistent nausea and gastric indigestion, which was intractable in nature requiring antiemetics and proton pump inhibitors. Troponin was found to be elevated at 2.5 ng/ml (normal reference <0.10 ng/ml); electrocardiogram showed ST-elevation in leads V2-V6 (Figure 1, panel B).

**Figure 2:**
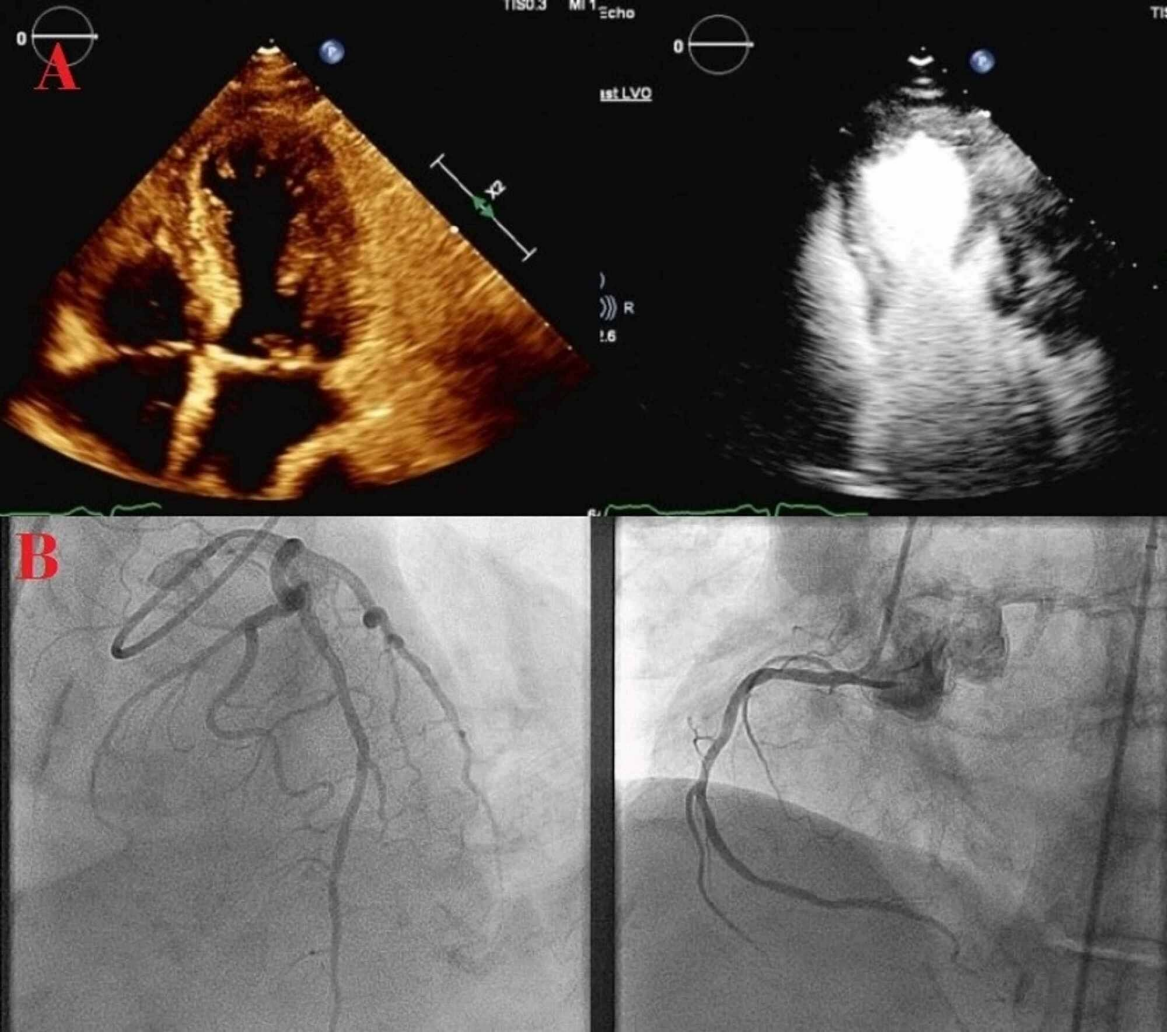
Radiological findings of takotsubo A) Echocardiogram 2D showing severe apical hypokinesis. B) Coronary angiogram showing minimal coronary artery disease (Left anterior descending artery on the left. Right coronary artery on the right.)

The echocardiogram revealed an ejection fraction of 35%-40% with severe apical hypokinesis (Figure 2, panel A). She underwent emergent cardiac catheterization for ST-segment elevated myocardial infarction (STEMI) and was found to have very mild coronary artery disease, unlikely to be responsible for her cardiac presentation (Figure 2, panel B), i.e., STEMI. The diagnosis of takotsubo cardiomyopathy accompanied by PRES was established. She reported immense psychological stress since the death of her brother; this was considered to be an attributor to both of her consecutive presentations.

## Discussion

Both PRES and TC disproportionately occur in postmenopausal females, linking shared etiological grounds [1]. Pathophysiologically, PRES may be related to disordered cerebral autoregulation (neuropeptide theory) and/or endothelial dysfunction (cytotoxic theory). Other mechanisms include increased systemic blood pressure (vasogenic theory), and T-cell activation causing increased endothelial permeability (immunogenic theory) [2]. Published data on TC suggest catecholamine surge playing a role in its pathophysiology [1]. In both patients with PRES and TC, clinical and radiological findings reverse. Perhaps, this occurs due to the settling of the trigger or acclimatization of the human body to the trigger.

Several central nervous system (CNS) disorders, such as ischemic stroke, seizures, subarachnoid hemorrhage, cranial trauma, and Guillain-Barre syndrome (GBS), are associated with TC, but the association between PRES and TC is rarely reported [1]. Previously published cases of concomitant PRES and TC are described in Table 1 [3-10]. A catecholaminergic surge due to physical or emotional stressors may cause direct myocardial injury leading to stress cardiomyopathy. Similarly, increased sympathetic tone due to a stressor may contribute to PRES due to the dysregulation of cerebral blood flow. In summary, autonomic and endovascular dysfunctions may be the fundamental changes contributing to both PRES and TC. Awatsu et al. recorded hypercatecholinemia due to stress responsible for both TC and PRES [10]. Among 13 cases of concomitant PRES and TC, 12 patients were females with one case being male (see Table 1), advocating hormonal factors. Potential initiators of concomitant PRES and TC in these patients were uncontrolled hypertension, physical and emotional stressors, acute illnesses, tyrosine kinase inhibitor (Lenvatinib), and GBS. The resolution of both the syndromes was almost 100% in these cases. PRES recurred in two cases while TC recurred in one case in one retrospective review of six cases reported by Summers et al. [6].

**Table 1:** Summary of cases presented with PRES and concomitant takotsubo cardiomyopathy (2007-2018) Abbreviations (Alphabetically): AMS: altered mental status, BP: blood pressure; Echo: echocardiogram; EKG: electrocardiogram; EF: ejection fraction; F: female; GBS: Guillain Barre syndrome; M: male; MRI: magnetic resonance imaging; MRA: magnetic resonance angiography, TC: takotsubo cardiomyopathy; PRES: posterior reversible encephalopathy syndrome, YO: year-old

Author, Yr.	Patient’s age and gender	Trigger	PRES	TC	Gap between PRES and TC	Outcome
Chae et al., 2018 [3]	58-YO-F	Lenvatinib for thyroid cancer	Clinical: Seizures, AMS. Radiological: MRI with multifocal hyperintensities of frontal, parietal, and occipital cortices. Vasogenic edema of frontal lobes. Treatment: Lacosamide, discontinuation of lenvatinib.	Clinical: Cardiogenic Shock. Radiological: Drop in EF on Echo from 62% to 36%, Hypokinesis of basal to mid-ventricular segments, and hyperdynamic apical segments. Treatment: vasopressor for shock, ACE inhibitor, and beta-blocker for ventricular dysfunction.	Within 2 days	PRES: Near-complete resolution of MRI findings TC: Recovery of EF. Survival: Death due to cancer
Grimaldi et al., 2017 [4]	69-YO-M	Not reported	Clinical: Left-sided hemiparesis, coma, seizures, elevated BP. Radiological: MRI with cortical, and subcortical vasogenic edema and posterior predominance. Cerebral angio showed no occlusions.	Clinical: comatose state, elevated troponin, transient LBBB. Radiological: Echo with apical akinesis and altered EF. Coronary Angio without stenosis but mid and apical hypokinesia.	Within 3 days	PRES: Resolution of vasogenic edema. TC: Normalization of EF, hypokinesis. Survival: Recovery in 3 months
Yadav et al., 2015 [5]	60-YO-F	Emotional stress	Clinical: vision loss, headache, nausea/vomiting, elevated BP Radiological: MRI with vasogenic edema of bilateral frontal and parietal lobes. MRA unremarkable. Treatment: strict control of BP	Clinical: Chest discomfort, elevated troponin, inferoposterior ST-elevation on EKG. Radiological: Echo with decreased EF and hypokinesis of basal anterolateral and inferolateral. Cardiac Cath with akinesis of basal, mid anterior, and inferior wall without coronary obstruction.	Within 1-2 days	PRES: Resolution of symptoms. TC: Resolution of STEMI. No fibrosis or infarction of cardiac MRI. Survival: Recovery in few days.
Summers et al., 2012 [6]	6 females, mean age of 64	Physical stressors, acute illnesses	Clinical: AMS, headache, seizures, visual changes. Radiological: vasogenic edema predominantly in the posterior circulation. Treatment: correct underlying cause, anti-seizures, control of BP.	Clinical: Dyspnea, chest pain, elevated troponin, ST-T wave changes on EKG. Radiological: Reduced EF and wall motion abnormalities on Echo. Treatment: Control BP, treat the underlying cause.	Within 1 day to 9 months	PRES: Improvement or near-complete resolution 1 patient with recurrence. TC: Resolution of low EF and reversal of wall motion abnormalities in all. 2 with recurrence. Survival: All patients recovered.
Fugate et al., 2009 [7]	82-YO-F	GBS	Clinical: uncontrolled BP, status epilepticus. Radiological: MRI with bilateral hyperintensities in occipital lobes and thalamus along with vasogenic edema. Treatment: Correct underlying cause, anti-seizures.	Clinical: Dyspnea, Hypotension. ST elevation. Radiological: Apical akinesis and EF of 30% on echo. Cardiac Cath without coronary stenosis.	Within 1 day	PRES: Near complete resolution in 2 weeks. TC: Improvement in EF in 2 weeks. Survival: Complete recovery.
Papanikolaou et al., 2009 [8]	47-YO-F	Uncontrolled BP due to renal failure	Clinical: Headaches, vision loss, and seizures. Radiological: MRI with white matter changes in the parietooccipital regions bilaterally. Treatment: Control BP, antiseizure.	Clinical: Cardiogenic shock, pulmonary edema, EKG with T-wave inversion in inferolateral leads. Radiological: Global left ventricular hypokinesis on echo. Treatment: vasopressors.	Few hours.	Survival: Resolution of the cardiac and neurological condition.
Banuelos et al., 2008 [9]	56-YO-F	Uncontrolled BP	Clinical: Headache, seizures, nausea, vomiting. Radiological: MRI with multifocal bilateral cortical and subcortical hyperintensities.	Clinical: Elevated troponins, nonspecific T-wave abnormalities. Radiological: Echo with EF of 40% and hypokinesis of the apical wall region with EF 40%. Cardiac Cath with non-significant coronary disease.	Within 3 days	PRES: Normalization of EF and reversal of wall motion abnormalities at 3 days TC: Resolution of MRI findings in one month.
Awatsu et al., 2007 [10]	45-YO-F	Stress, elevated catecholamines	Clinical: Nausea, vomiting, vertigo, visual problems, and seizures. Radiological: MRI with multiple T2 lesions in occipital lobes.	Clinical: Chest discomfort, EKG with ST-elevation. Radiological: Hypokinesis in left ventricular apex on echo.	Within 1 day.	Outcome unknown

## Conclusions

The presentation of these two entities in a single patient is rare. Our case further validates the theory of “heart and brain connection,” with emotional stress leading to a catecholamine surge - potentially a shared mechanism connecting the two disorders. It is highly likely that both of these syndromes may have shared pathophysiology and common precipitating factors that need to be further explored and determined.
